# Biosensors for the Detection of Antibiotics in Poultry Industry—A Review

**DOI:** 10.3390/bios4040472

**Published:** 2014-11-21

**Authors:** Nawfal Adam Mungroo, Suresh Neethirajan

**Affiliations:** BioNano Laboratory, School of Engineering, University of Guelph, Guelph, ON N1G 2W1, Canada; E-Mail: nmungroo@uoguelph.ca

**Keywords:** analytes, antibiotic resistance, biosensors, maximum residue limits (MRLs), surface plasmon resonance (SPR)

## Abstract

Antibiotic resistance is emerging as a potential threat in the next decades. This is a global phenomenon whereby globalization is acting as a catalyst. Presently, the most common techniques used for the detection of antibiotics are biosensors, ELISA and liquid chromatography—mass spectrometry. Each of these techniques has its benefits as well as drawbacks. This review aims to evaluate different biosensing techniques and their working principles in order to accurately, quickly and practically detect antibiotics in chicken muscle and blood serum. The review is divided into three main sections, namely: a biosensors overview, a section on biosensor recognition and a section on biosensor transducing elements. The first segment provides a detailed overview on the different techniques available and their respective advantages and disadvantages. The second section consists of an evaluation of several analyte systems and their mechanisms. The last section of this review studies the working principles of biosensing transducing elements, focusing mainly on surface plasmon resonance (SPR) technology and its applications in industries.

## 1. Introduction

Antibiotics have been used extensively in the animal food industry since the early 1940s. The practice of using antibiotics in veterinary medicine has revolutionised the industry. Antibiotics are mainly utilized for the treatment of infection as a means for disease prevention (prophylaxis), such as treating respiratory and enteric infections during the early stage of the animals’ lives or for the treatment of bacterial infections, such as mastitis in the adult stage. Additionally, antibiotics are used as feed in sub-therapeutic concentrations to enhance growth [1]. This practice proves to be highly profitable as more flesh/meat can be obtained from animals receiving such treatment. The extensive use of sub-therapeutic dosages administered in concentrated animal feeding operations (CAFOs) increases the number of animals with antibacterial resistant properties. As a result, an imbalance in the ecology is created, resulting in the spread of numerous resistant genes amongst other animals and humans [2]. This is alarming for humans in particular, since resistance to antibiotics signifies reduced drug effectiveness. This results in the reduced efficiency of antibiotic treatment; it may become impossible to treat infections. Other negative impacts include treatment failures and a rise in the rates of morbidity and mortality as well as costs to society. One important example of the dangers of anti-bacterial resistance is illustrated in the outbreak of *Salmonella* in the city of Montevideo, USA, causing the infections of 245 individuals between July 2009 and March 2010 [3]. Unfortunately, this crisis is not restricted to North America; rather, it continues to affect the world in a significant way. For instance, there have been reported deaths of more than 25,000 individuals in the European Union (EU) alone [1].

The fast propagation of resistant bacteria is exemplified by the results of a recent Canadian study showing a strong relationship between the pathogen *Salmonella enterica serovar Heidelberg* and the commensal *E. coli* from retail chicken and human infections [4]. The main individuals at risk are farm workers, slaughterhouse employees and veterinarians since they are in direct contact with the animals. Infected individuals further act as carriers and spread the pathogens throughout their surroundings [4]. Evidence indicates that the risk of poultry workers being affected by gentamicin-resistant *E. coli* is 32 times higher than that of non-poultry workers. Fifty per cent of poultry workers are affected by the colonization of gentamicin-resistant *E. coli* as compared to only 3% of non-poultry employees [5]. It is to be noted that this phenomenon not only applies to the poultry industry but can also extend to other animal industry, such as pigs and cattle. Additionally resistant bacteria can also be transmitted through the consumption of animal products [4]. This type of transmission is highly significant given that it targets a larger population including infants, children and senior citizens. This transmission route is more complicated to trace as the product is exposed to resistant bacteria during all stages of food processing as well as to different animals [4]. With the mass production of imported products catalyzed by globalization, anti-bacterial resistance will not remain a local problem but may negatively impact the world at a very fast pace.

To reduce the risk of anti-bacterial resistance, the European Union instigated a “precautionary principle” model by banning certain antimicrobial growth promoters [5].

For those antibiotics that are not banned, maximum residue limits (MRLs) of antibiotics have been set by both European countries and the United States to ensure the safety of consumers. According to the European Union’s definition, the MRL is the maximum legally acceptable amount of pharmacologically active substances (whether active principles, excipients or degradation products) and their metabolites in foodstuffs originating from animals. The MRL is calculated with reference to the Acceptable Daily Intake (ADI), which includes a large safety margin in the calculation. For instance, the ADI for meat is about 500 grams per person. Those regulations are contingent upon the withdrawal period, which is the time period between the last dose of any pharmacologically active substance or of antibiotics in particular administered to the animal and the time at which the residue level in tissues (muscle, kidney, skin/fat, liver) or products (milk, honey, eggs) is lower than or equal to the MRL. Withdrawal periods promote consumer safety by certifying that the MRL is not exceeded; they also ensure reinforced safety in case the MRL is not met [6]. Several techniques have been developed in order to accurately and practically test whether MRLs have been met in meat and for performing the tests necessary in order to determine safe MRLs to be used in policy making. This review aims to evaluate different biosensing techniques and their working principles in order to accurately, quickly and practically detect antibiotics in chicken muscle and blood serum.

The review is divided into three main sections: namely, an overview of biosensors, a section on biosensor recognition and a section on biosensor transducing elements. The first segment provides a detailed overview on the different available biosensing techniques and their respective benefits and drawbacks. The second section consists of evaluating several analyte systems and sensing mechanisms. The last section of this review focuses mainly on surface plasmon resonance (SPR) technology and its applications in industries.

### Antibiotics and Food Safety

There exist several families of antibiotics: beta-lactams (penicillins and cephalosporins), chloramphenicols, tetracyclines, macrolides, lincosamides, spectinomycin sulphonamides, nitrofurans, trimethoprim, nitroimidazoles, polymyxins, quinolones and macrocyclics (ansamycins, glycopeptides and aminoglycosides) [7].

**Figure 1:**
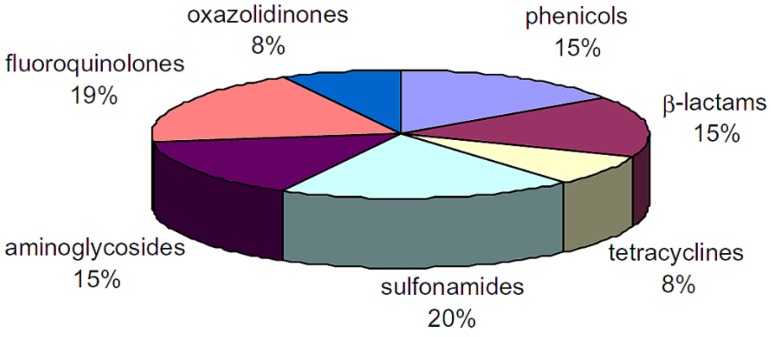
Classification of antibiotic families present in food [7].

Fluoroquinolones (FQs) and sulfonamides (SAs) are the most popular antimicrobial agents used for the promotion of growth and for the treatment of infections due to their broad spectrum against both Gram positive and Gram negative microorganisms [8]. Figure 1 illustrates the distribution of the antibiotic families present in food. The Canadian MRL values for sulfonamides, penicillin G, streptomycin and tetracycline are 0.1 ppm (cattle muscle), 0.05 ppm (chicken muscle), 0.5 ppm (chicken muscle), and 0.2 ppm (chicken muscle) respectively [9]. As can be seen, there are different MRL requirements and families of antibiotics, which makes their detection more complicated. Hence, there is a dire need to qualitatively and quantitatively detect antibiotics in poultry meat.

## 2. Antibiotics Detection Techniques

With the emerging concern regarding anti-bacterial resistance, several analytical methods have been developed to determine levels of antibiotic residues in meat products. The analytical methods for antibiotics detection can be divided into two groups, namely confirmatory and screening [7].

Confirmatory methods depend on the properties of liquid chromatography alongside mass spectrometry (LC-MS) in the determination of the concentrations of analyte. Other detection methods include a combination of liquid chromatography and UV (LC-UV) or electrophoresis based approaches [7].

**Figure 2:**
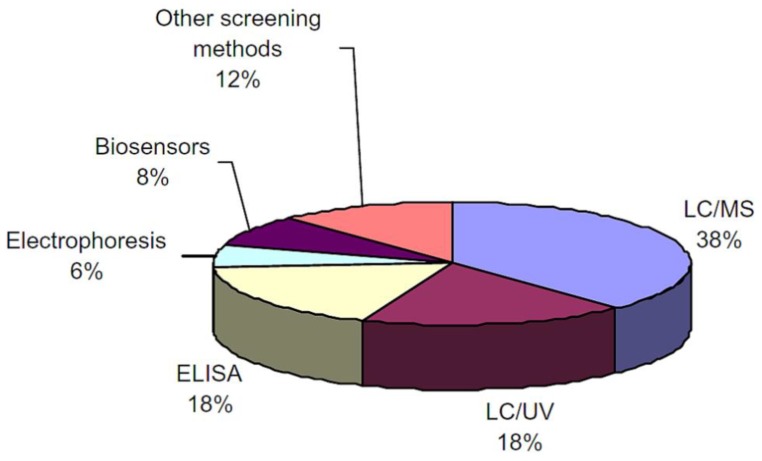
Analytical methods classification used for antibiotics determination in food [7].

Screening methods are primarily used to obtain semi-quantitative measurements. This approach is viable because of the low possibility of false-positive data, easy operation, quick analysis period, cost effectiveness and good selectivity. Figure 2 illustrates the distribution of analytical methods used for antibiotics determination in food [7]. Biosensors use a semi-quantitative approach, which makes them a very practical solution in the large-scale detection of antibiotic residue in animals including at farms and slaughterhouses. However, biosensors have limitations with respect to both stability and sterilization. Table 1 illustrates the comparisons between the most common techniques used for antibiotics detection.

**Table 1:** Comparison of techniques for the detection of antibiotic residues in meat.

Detection Method	Principle	Advantages	Drawback
**Enzyme-Linked Immunosorbent Assay (ELISA)**	- Most microbial species have a minimum of one unique antigen, which can be utilized for the generation of monoclonal antibodies. The antibodies and antigens are effective and highly specific tools for diagnosis. - ELISA is a common serological technique for the detection of antigens and antibodies. ELISA is divided into two forms of assays: direct ELISA and indirect ELISA. Direct ELISA involves the use of monoclonal antibodies for the detection of a specific antigen. Indirect ELISA involves detecting a specific antibody in a sample, such as serum [10].	- High sensitivity and broad specificity [10] - Detection of multiple samples at one time in a short time period ensuring practicality and convenience in terms of large scale testing [11] - Screening of a large number of small-volume samples [11]	- Time-consuming due to sample pre-treatment and clean-up [12] - Not practical for quick detection [12] - Expensive [12] - Detection is not real-time [12]
**Biosensors**	- An instrumentation that comprises two key elements: a transducing device and a recognition element. - The transducer is utilized to detect any contacts between the affinity-pairing partners by converting the biological response into useful electrical signals [13].	- Measure nonpolar molecules that are not receptive to most devices [13] - High in specificity as a result of immobilized system inside them [14] - Quick (short response time) and long-lasting/duration control [14] - Practical and real-time application for industry use [13]	- Cannot undergo heat sterilization due to the possibility of denaturation of the biological element [13] - Stability of biological material (cell, antibody, tissue, *etc.*) is contingent upon natural properties of the molecule under environmental conditions (pH, temperature, ion) [13] - Possible risk of contaminations of cells in biosensors through diffusion of substances across membrane [13] - Restriction due to the size of the transducer element within the biosensors [7]
**Liquid Chromatography-Mass Spectrometry (LC-MS)**	The LC-MS coupling is an effective system whereby the mass spectrometry component functions by transforming the ionised (charged) state of molecules using the mass-to-charge ratio. There are several methods of LC-MS, including the electrospray ionisation source, direct injection methods and mobile phase [14].	Highly sensitive and able to handle complex mixtures [14]	- Not practical, time-consuming, low throughput and expensive due to sample and clean-up procedures [11] - Require qualified personnel and costly equipment [11]

## 3. Overview of Biosensors

### 3.1. Definition of Biosensors

A biosensor is an instrumentation that comprises two key elements in close proximity: a transducing device and a recognition element with a supporting material. The recognition element consists of two affinity-pairing partners (antibody/antigen, enzyme/substrate, receptor and its specific ligand, or living cells and an analyte that binds specifically to them), one of which is immobilised [13].

**Figure 3:**
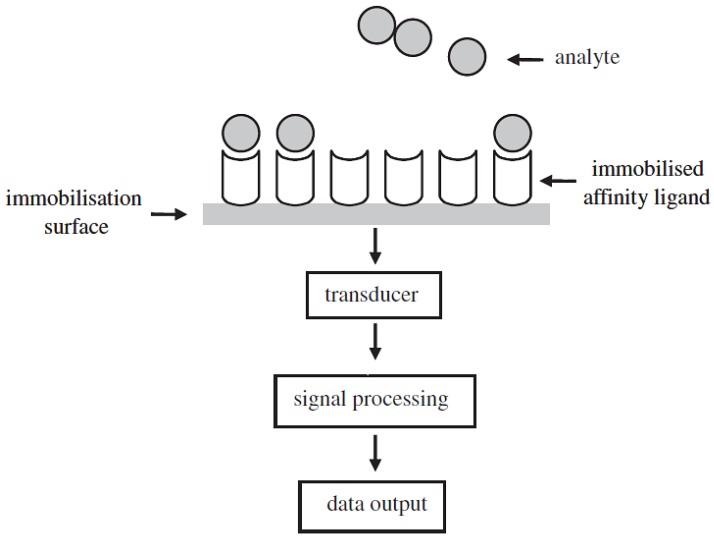
Schematic representation of the configuration of a biosensor [13].

The transducer is utilized to detect any contacts between the affinity-pairing partners by converting the biological response into useful electrical signals. A processor then transforms the electrical signal [13] for interpretation. The basic configuration of a biosensor is illustrated in Figure 3.

The simplicity of the mechanism of biosensors as compared to other detection techniques makes the detection of antibiotics with biosensors relatively fast, accurate and easy to implement. The primary limitation of biosensors is the uncertainty of the biological sensing element. For instance, the sensing mechanism may be affected by duration of use, type of molecules, and/or environmental factors (pH, temperature and ionic strength). Another restriction of biosensors is the transducer size within the biosensors [7].

### 3.2. Classification of Biosensors

**Figure 4:**
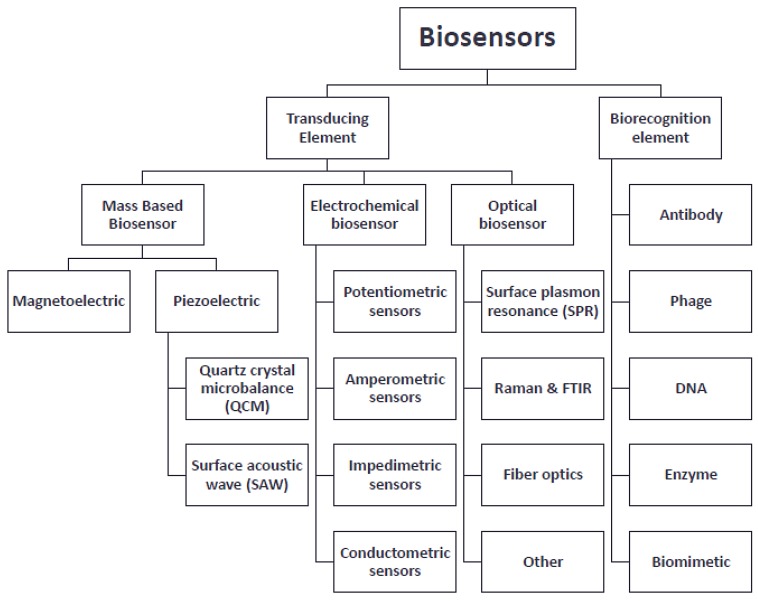
Schematic representing the classifications of biosensors. (Contents adapted from [14]).

Biosensors are grouped according to their signal transduction and their bio-recognition elements as illustrated in Figure 4 [14].

## 4. Types of Biosensors

Biosensors are generally divided into three main types, namely: (1) electrochemical biosensors; (2) mass-based biosensors; and (3) optical-based biosensors. The working principles of each of the three biosensors are different and can thus be implemented in a variety of applications. The following section outlines different types of biosensors and offers a brief description of their working mechanisms.

### 4.1. Electrochemical Biosensors

Electrochemical sensors (potentiometric, amperometric, impedimetric and conductometric) measure the electrical potential difference (electromotive force, EMF) caused by an interaction between an analyte and the membrane/sensor surface. There is proportionality between the electrical potential difference and the logarithm of the electrochemically active concentration of the material. Electrochemical biosensors provide high specificity with rapid analysis at a reasonable cost [14]. There exist four types of electrochemical biosensors, namely potentiometric biosensors, amperometric biosensors, conductometric/capacitive biosensors and impedimetric biosensors [13].

▪ Potentiometric-based biosensors sense changes in pH and ion concentrations upon the antigen/antibody interaction.
▪ Amperometric-based biosensors sense the difference in current potentials during redox reactions when antigen/antibody pairing occurs.
▪ Conductometric/capacitive biosensors sense the change of electrical charge in a solution under constant voltage. This method is not recommended because of poor signal-to-noise ratio.
▪ Impedimetric-based biosensors sense changes in impedances upon antigen/antibody interaction.

### 4.2. Mass-Based Biosensors

**Figure 5:**
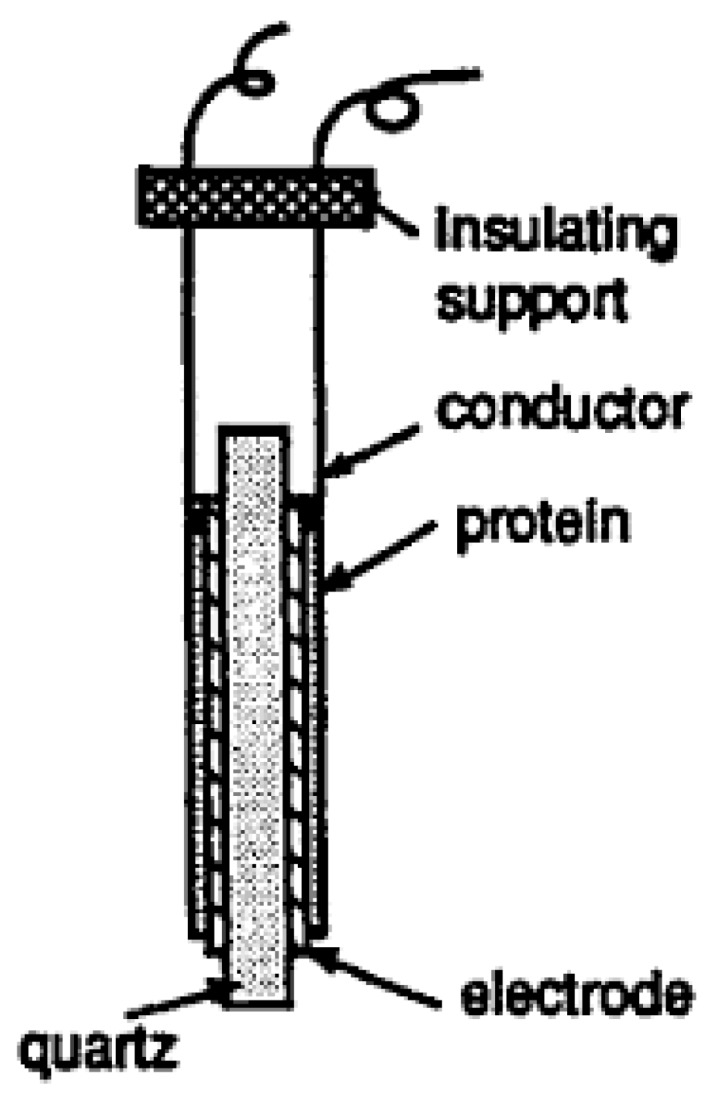
Quartz crystal microbalance (QCM) [15].

Piezoelectric biosensors are considered as mass-based biosensors. Piezoelectric biosensors produce an electrical signal when a mechanical force is applied. They can be used for label-free detection of specific nucleic acid sequences for antibiotics detection in food. The most common type of piezoelectric biosensor is the quartz crystal microbalance (QCM) model. It uses specific oligonucleotides, which are restrained on the surface of the quartz crystal and inputted in a solution containing potential target nucleic acids, as illustrated in Figure 5. Upon interaction between the target nucleic acids and its complementary oligonucleotide, there is an increase in the mass of the piezoelectric biosensor, which is proportional to the decrease in the resonance frequency of the quartz oscillation. The main advantages of using piezoelectric biosensors include real-time output, cost effectiveness and practicality for large-scale use. The drawbacks are lack of specificity and sensitivity as well as the possibility of interference at the sensor surface [15].

QCM operates at its optimal level if used with a molecular imprinting procedure. As the name states, this technique depends on molecular recognition whereby polymerization occurs around the target molecule. Consequently, this approach ensures that specific cavities are developed in the cross-linked polymeric matrices. This novel method can be used on polymeric nanofilms on the QCM sensor chip using a molecular imprinting approach, resulting in an enhanced real-time sensing platform [16].

### 4.3. Surface-Enhanced Raman Scattering (SERS)

**Figure 6:**
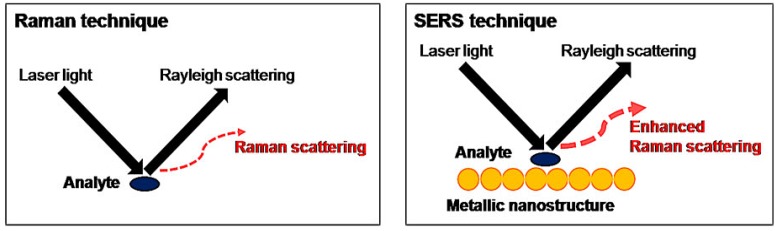
The principles behind Raman and SERS techniques [17].

A recent development in antibiotics detection is the surface-enhanced Raman scattering (SERS) technique. The particularity of the SERS system is the addition of a metallic nanostructure to enhance weak signals and reduce the risk of fluorescence interference, as illustrated in Figure 6 [17]. The benefits of SERS can be explained using the principles of electromagnetic enhancement and chemical enhancement. In electromagnetic enhancement, an extensive electromagnetic field is present, promoting the excitation of localized surface plasmon resonance. As a result, there is an improvement in weak signals and thus higher sensitivity is obtained. Chemical enhancement is achieved by increasing the probability of a Raman transition when there is absorption of molecules on the roughened surfaces of the metallic nanostructure.

Gold (Au) and silver (Ag) colloids of diameters 10 to 200 nm are the most common active substrates used in SERS’s nanostructure. Gold and silver are highly practical since they are inexpensive, easy to manufacture on a large scale and widely available. However, the main issue with both metals is poor performance in terms of reproducibility. This is due to the non-uniform aggregation of nanoparticles on the solid surface after the drying-up process. To remedy this problem, colloid-based substrates have been introduced. These include synthesis of multicomponent nanoparticles, such as Ag-coated (ferriferous oxide-core silica shell) composite microspheres. Subsequently, the sensitivity of the device has increased and the following antibiotics have been successfully detected: tetracycline, furadantin, furaltadone, ciprofloxacin, enrofloxacin and chloramphenicol [17]. Despite its numerous advantages, SERS technology is extremely costly with Raman instruments costing between USD 80,000 and 200,000. This limits the application of SERS to indoor use. Hence, there is a need for the development of a portable SERS device for on-site detection. The integration of SERS with other separation techniques is an additional possibility to enhance the practicality of SERS.

### 4.4. Optics Biosensors

**Figure 7:**
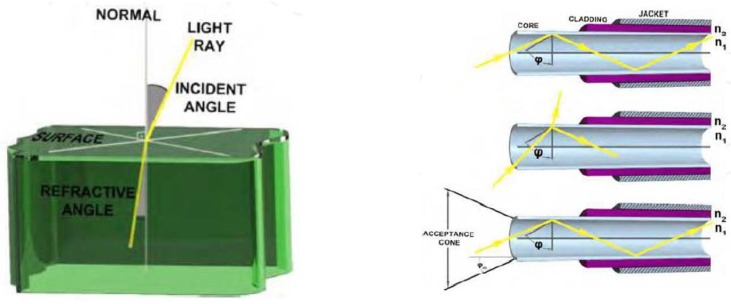
An example of fibre-optic biosensors (adapted from [14]).

Fibre-optic biosensors are considered as optical biosensors since they use the reflective properties of light to sense numerous analytes simultaneously. Fibre-optic biosensors consist of a core (n_1_), cladding (n_2_) and jacket (n_3_), each with a different refractive index, as illustrated in Figure 7. The core and cladding are critical for light transmission. Fibre-optic biosensors are excellent for their high specificity, practicality (due to the absence of a reagent in contact with the optical fibre), and cost effectiveness. Some of the drawbacks of fibre-optic biosensors include a short lifespan of reagents under incident light and slow response time as a result of the diffusion of analytes [14].

#### 4.4.1. Absorbance Fibre-Optic Biosensors

This type of biosensor measures the transmitted and scattered light through the fibre and then measures the absorbance values using the Lambert Beer Law as illustrated in Equation (1).

Lambert Beer Law used for absorption

(1) $$A=\log\left(\frac{I_{o}}{I}\right)=\text{є}.\left[c\right].l$$

where

*A* = Optical Absorbance.
*I_o_* = Incident light intensity.
*I* = Transmitted light intensity.
є = Molar absorption coefficient.

#### 4.4.2. Fluorescence Fibre-Optic Biosensors

The use of fluorescence fibre-optic biosensors leads to the detection of signals by transferring the excitation light through an optical fibre; the emitted light is then measured via a detector. The change in fluorescence intensity is related to the analyte concentration. Fluorescence fibre-optic biosensors are a better option when compared to absorption sensors due to their ability to measure low concentrations of analyte [14].

#### 4.4.3. Luminescence Fibre-Optic Biosensors

Luminescence fibre-optic biosensors detect the light emission from a chemical reaction whereby excited species return to the ground state. Luminescence fibre-optic biosensors can further be classified into chemiluminescence and bioluminescence [14]. Likewise, there are two types of bioluminescence biosensors: Adenosine triphosphate (ATP) bioluminescence and bacterial bioluminescence [14].

##### ATP Bioluminescence

The use of bioluminescence in performing tests to determine antibiotics levels consists of swabbing a sample and mixing it into a solution of luciferase/luciferin (enzyme/substrate). A summary of the reactions is illustrated below:


luciferin + ATP => luciferyl adenylate + inorganic pyrophosphate (PPi)

Light is produced once luciferyl adenylate reacts with oxygen. Other products of this reaction include oxyluciferin and AMP [14].


luciferyl adenylate + O_2_ => oxyluciferin + AMP + light

The main benefits associated with bioluminescence include high sensitivity, quick detection and easy implementation in portable devices [14].

The drawback primarily involves the non-specificity of ATP assays as the latter is found in all living cells. To solve this problem, bioluminescence sensors are coupled with other sensors and detection techniques [14].

##### Bacterial Bioluminescence

**Figure 8:**
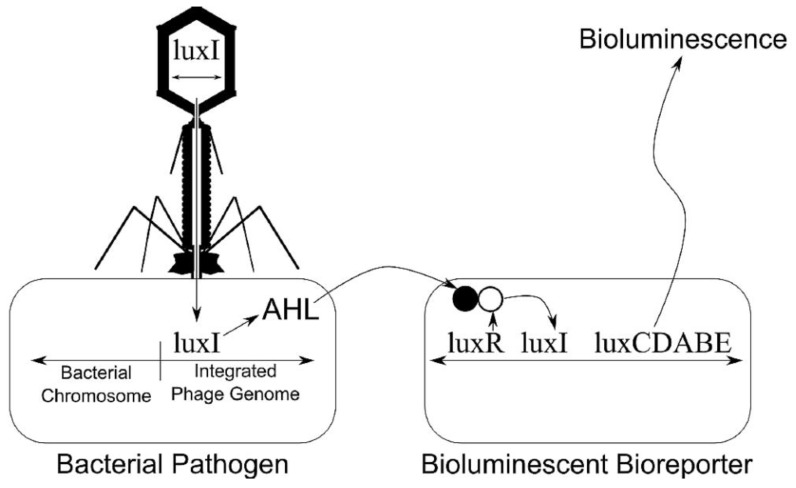
The principle of bacterial bioluminescence [15].

The principle behind bacterial bioluminescence involves the transfer of the lux gene into the host bacterium in the case of an infection. The resultant reaction causes bioluminescence to occur; luminometers are then utilized to detect the emission of light [14], as illustrated in Figure 8.

## 5. Design Strategies of Fluorescent Biosensors

Fluorescence-based techniques are the most common techniques currently being implemented in biomolecular imaging due to their excellent sensitivity and selectivity, satisfying spatial and temporal resolution and cost effectiveness. Fluorescent biosensors are designed by synthetic receptors or biological macromolecular receptors, specifically proteins and aptamers. The designing of a fluorescent biosensor requires two key steps. The first step entails searching for a macromolecular receptor with optimal affinity and specificity to the target; the second step consists of integrating the receptor component and the signal transduction function such that an interaction between the macromolecule and the target induces a signal that can be captured by the transducer. However, an additional step consisting of integrating moieties, such as auto-fluorescent protein (AFP) or synthetic fluorophore, must be added since biological receptors lack signal transducing capabilities [18].

### Auto-Fluorescent Protein (AFP)-Based Biosensors

Common AFPs, such as green fluorescent protein (GFP), can be found in jellyfish. The mechanism of AFPs consists of the exhibition of a spontaneous fluorescent emission in cells through the autocatalytic formation of the chromophore after translation. This procedure allows no interference of fluorescence properties or damage to cells since AFPs can be endogenously expressed through transfection of the plasmid DNA [18]. AFP-based biosensors are crafted according to two separate designs; they are termed analyte-sensitive sensors and conformation-sensitive sensors.

#### Analyte-Sensitive Sensors

The design of analyte-sensitive sensors is based on AFP variants, whose fluorescent properties are directly affected by the interaction between a target molecule and a chromophore moiety in AFP. Initially, pH and halide-sensitive AFP variants have been developed exploiting the intrinsic pH sensitivity of GFP mutants [18].

#### Conformation-Sensitive Sensors

Conformation-sensitive sensors operate when there is a conformational alteration of the receptor associated with the ligand-binding event, leading to the creation of a fluorescent signal response from AFPs. This conformational alteration characteristic ensures more versatility as compared to analyte-sensitive sensor given that it can sense more varieties of protein [18].

## 6. Biosensor Recognition

Two primary factors dictate the performance of biological recognition elements (ligands) in biosensors. The first factor is the affinity and specificity of the ligands, which is determined by the ligand dissociation constant (Kd). The second factor involves the constrained shelf lives of the biorecognition elements [13].

The two main techniques for recognizing antibiotics are the use of aptasensors and the use of antibody-mediated binding processes. Aptasensors can be defined as RNA- and DNA-immobilized aptamers (oligonucleic acids), which cause the binding of the analyte of interest to their respective 3D structure through ionic interaction, van der Waals forces or hydrogen bonds resulting in measureable signals. Aptamers are a preferred option for several applications due to their high binding affinity, easy labelling and high stability [19]. From a manufacturing standpoint, aptamer modifications and production are easy given that the molecules are resistant to degradation and denaturation.

**Figure 9:**
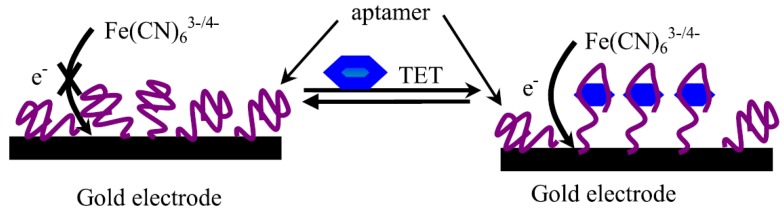
Binding of Tetracycline with Tet-binding Aptamers [20].

One of the key developments in aptasensor technology is the combination and screening of tetracycline (TET)-binding aptamers via isothermal titration calorimetry, which is illustrated in Figure 9. The results of Chen *et al.* [20] showed evidence of an increase in response time, sensitivity and specificity. Additionally, no sample pre-treatment, such as washing or labelling is required, making the detection process direct, quick and practical [20]. However, aptamer-based biosensors are limited in their practicality due to the challenges in synthesizing aptamers with appropriate affinity and selectivity. One possible solution involves synthesizing aptamers that are resistant to cell degradation [20]. Another novel approach involves integrating aptamers with cantilever sensors. The principle behind this integration is to make use of the interaction between the immobilized receptor molecules, located on one side of the cantilevers, with target molecules. The binding of the receptor to the target molecule causes a change in surface stress between the cantilever’s functionalized and non-functionalized sides, ultimately resulting in a bending of the device. The bending of the cantilever is transduced into nanomechanical signals for interpretation. As a result, aptamer-based cantilever sensors provide excellent sensitivity, superior dynamic response, extended reliability and easy miniaturization [21].

The second mechanism for the recognition of antibiotics involves antibiotics detection using antibody-mediated binding processes. This type of binding can be performed at the surface of the sensor for direct detection of the antibiotics or by inverting the assay and detecting the binding of antibody-spiked samples onto immobilized antibiotics in the context of a competitive assay [19].

Apart from the two principles discussed above, the use of enzymes and the use of functionalised gold nanoparticles are new and innovative methods in the field [20]. The introduction of gold nanoparticles in biosensors ensures improved biocompatibility, sensitivity and easier preparation. Gold nanoparticles can be used as a labelling agent for biological receptors, such as DNA, enzyme antigens/antibodies and other biomolecules [22]. Recent applications of gold nanoparticles in antibiotics detection include utilizing UV-vis absorbance spectrometry as a platform. The size, shape and local dielectric surrounding of gold nanoparticles dictate the resonance wavelength; they can thus be used for the detection of a variety of antibiotics families by simply adjusting those parameters [22]. These same features of gold nanoparticles also make it possible to build a colorimetric sensor for aptamers, peptides and ligands. A colorimetric-based approach is efficient for use in salts and other interferences for fast-paced detection. However, colorimetric-based sensors do not provide any information about the different families of tetracycline or other antibiotics [23].

## 7. Biosensor Transducing Element: Surface Plasmon Resonance (SPR)

**Figure 10:**
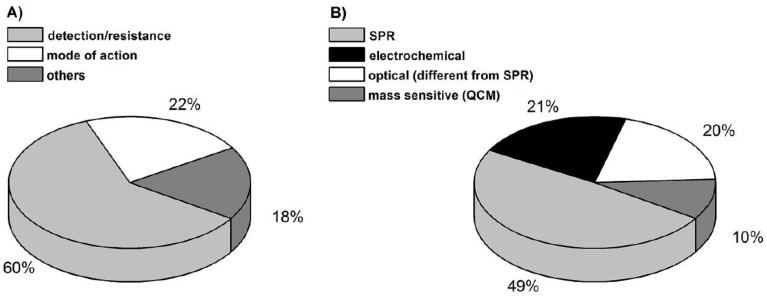
Distribution of detection techniques for antibiotic residues in the food industry (Reproduced with permission from K. Reder-Christ and G. Bendas [19]).

The second type of optical biosensor is called surface plasmon resonance (SPR). Forty-nine per cent of the 60% of biosensors used for the detection or quantification of antibacterial or antimicrobial residues in the food industry operate under the SPR technique, as illustrated in Figure 10 [19]. It is to be noted that the use of SPR is about 2.5 times greater than that of any other optical biosensing method. SPR is the primary choice due to its robustness and high sensitivity as well as its practicality in terms of rapid performance, reliability and high cost effectiveness [13].

### 7.1. SPR Transducing Recognition

**Figure 11:**
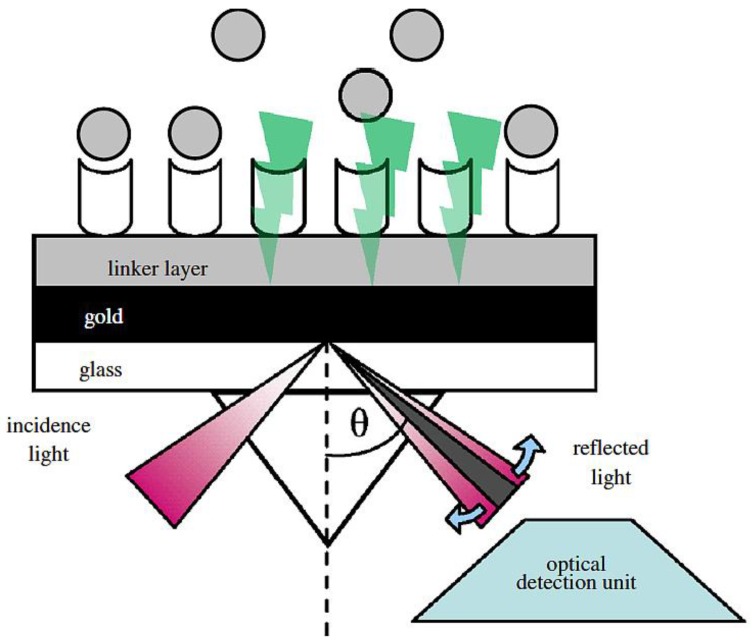
Schematic of working principle of surface plasmon resonance (SPR) biosensor [13].

Surface plasmon resonance is an optical transducer element and is commonly used in food safety. The principle behind the mechanics of SPR is illustrated in Figure 11. Initial rays of polarized light strike a thin conducting film at the interface between two transparent media with different refractive indexes. Some of the initial rays of polarised light (photons) are absorbed by the conducting film while the remaining diminished light energy is reflected at a specific angle (SPR angle) due to different refractive indexes. The interaction of the affinity partners causes a change in the reflective index. The SPR biosensors measure the variations in mass concentrations that result from changes in refractive indexes [15]. The changes of SPR angle over time are represented on a sonogram. The unit signal of the SPR is defined as a resonance unit (RU), which is equivalent to approximately 1 picogram·(pg)/mm^2^ of material bound of surface area [24].

### 7.2. Biorecognition Element of SPR

There are two main SPR assay formats for the detection of small chemical compounds. Smaller analytes are quantified using a competitive or inhibition assay. Competitive assays consist of a conjugated analyte being added to the sample and the latter competes to bind with the surface couple ligand. Inhibition assays consist of a fixed concentration of analyte binder that is added to the sample; the remaining free binding agent is captured by immobilizing analytes on the sensor chip [24].

**Figure 12:**
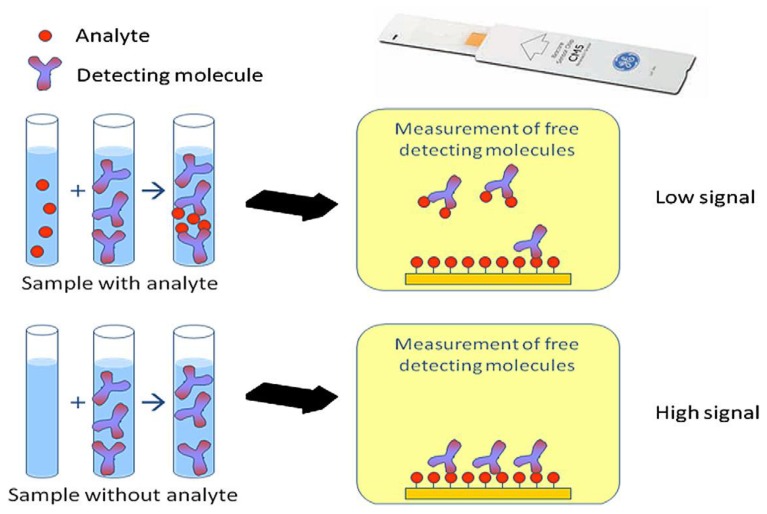
Schematic showing the principle of immobilization on a biosensor [24].

The design of SPR screening assays is based upon a competitive inhibition assay model whereby antibodies are used as biological recognition element. The preparation involves injecting a known concentration of drug-specific antibody mixed with the sample over the surface sensor chip with a drug derivative (pesticide, contaminant) immobilized onto it. In the absence of antibiotic residue in the sample, the antibody is able to bind to the immobilized antigen on the sensor surface, leading to the maximum value in SPR response units (RU). If antibiotic residue is present in the sample, the antibody binds to the antibiotic, inhibiting antibody binding to the derivative on the sensor chip surface and leading to a lower RU value. An increase in the concentration of the drug in the sample causes an increase in inhibition level and a lower SPR response, as illustrated in Figure 12. When free analytes are used, the free and immobilized analytes compete for the antibodies [13]. In the inhibition assay, a fixed concentration of analyte binder is added to the sample and any of the binders that remain free in solution are captured by immobilized analytes or analyte analogues on the sensor chip.

However, assays need to be optimized with regard to different parameters, such as antibody concentration, incubation time, pH and molarity of extraction/dilution, antibody/sample ratio and loading buffer, contact time, flow rate, and regeneration solution [24]. In short, the recognition element is equal in importance to the transducing element. Optimizing the recognition of antibiotics increases the sensitivity and accuracy of the detection system by ensuring detection of more families of antibiotics as well as a low concentration of analytes.

**Table 2:** Surface plasmon resonance applications in the detection of antibiotics.

Antibiotics	Matrix	Biosensor Type	LOD (Limit Of detection)	Sample Pre-Treatment & Assay time	References
**Aminoglycosides: Streptomycin, dihydrostreptomycin**	Pig muscle	Optical biosensor—SPR	70 ng/g muscle	Buffer + adjustment pH, kidney and muscle: 3% trichloroacetic acid or 3.6% HClO_4_ or 0.2 M Na_2_HPO_4_ at pH 9.2 4 h at 45 °C in tube (Accusphere test)	[25]
**Quinolones: Norfloxacin, enrofloxacin, sarafloxacin, flumequine, ciprofloxacin, difloxacin**	Chicken muscle	Optical biosensor—dual SPR + LC electrospray time-of-flight MS	LC50: between 2.7 and 5.9 ng/g for multi-FQs and 3.8 ng/g for flumequine	Homogenize with water, filtration of supernatant, ultrafiltration 3 h at 67 °C in tube (Farm test)	[26]
**Macrolides/Fenicol: Fenicols: CAP, CAP glucuronide**	Poultry muscle	Optical biosensor—SPR	0.005 ng/g for poultry muscle	(PBS) + ethyl acetate then evaporation supernatant 4 h at 45 °C in tube (Accusphere test)	[27]
**Tetracyclines: Chlortetracycline, oxytetracycline, tetracycline, doxycycline and their 4-epimer metabolites**	Poultry muscle	Bioluminescent biosensor bacterial	5 ng/g for doxycycline, 7.5 ng/g for chlortetracycline, and 25 ng/g for (oxy)tetracycline	Heating, centrifugationto obtain meat fluid sample. 3 h at 67 °C in tube (Farm test)	[28]
**Sulfonamides: Sulfamethazine, sulfisoxazole, sulfachlorpyridazine, sulfachlorpyrazine, sulfamerazine, sulfadiazine, sulfatroxazole, sulfathiazole**	Chicken serum	Optical biosensor—SPR	10 ng/mL for sulfamethazine	None	[29]

The practicality and efficiency of SPR detection are notable, as illustrated in Table 2. SPR technology can be used to detect the major families of antibiotics, such as quinolones, macrolides/Phenicol, tetracyclines and sulfonamides in chicken muscle. However, there is room for expansion, moving for instance toward the detection of aminoglycosides in chicken muscle instead of pig muscle or the detection of other antibiotic families, such as phenicols amongst others. Additional modifications could be made, such as eliminating the sample pre-treatment step to ensure a faster detection process.

## 8. Microfluidics and Biosensors

Microfluidics technology is a useful and increasingly popular tool used for the detection of analytes in several disciplines including food safety. Microfluidics technology is advantageous because of its ability to handle small sample sizes, which can be translated to less reagent wastage and quick detection. Additionally, microfluidics is utilized for point-of-care (POC) operations on sites due to its high level of efficiency and its reduction of the risk of cross-contamination. The new microfluidics technology has shifted into paper-based chips since there is no requirement for sample pre-processing. Moreover, there is no need for a power source to control fluid movement since the flow motion is influenced by capillary forces. The introduction of thermoplastic chips has also proven a viable option in terms of POC practices due to the disposability and cost effectiveness of the chips [30].

The integration of microfluidics and fluorescent labels ensures a decrease in sample volume, which further results in a reduction in background signal noise. This ultimately increases sensitivity and signal-to-noise ratio, Raman scattering and Rayleigh stray light. However, the chip material must possess microscopy-compatible characteristics, such as being non-adsorbent to the molecules and having low auto-fluorescence.

Microfluidics can also be incorporated with fluorescence resonance energy transfer (FRET) for the analysis of DNA. Microfluidics allows enhanced spatial and temporal resolution as compared to traditional fluorescently labelled molecules, as well as easy differentiation between hybridizing and non-hybridizing DNA oligomers. However, FRET-based techniques usually face problems of photo bleaching and pH sensitivity. To solve these issues, quantum dots (QD) are utilized for improved stability and signal. QDs’ ability to be bright and their extensive emission wavelength, which can be adjusted by altering their size and composition, make it possible to integrate them with microfluidics for antibiotics detection. The combination of QD + Microfluidics or QD + FRET or QD + FRET + Microfluidics ensures an amplified signal when sensing DNA and when detecting molecular size, binding and orientation. Gold nanoparticles are frequently used in antibiotics detection due to their easy conjugation to biomolecules, versatility in detection techniques and low toxicity. According to a study performed by Rivet, C *et al.*, the combination of gold nanoparticles and microfluidics resulted in a sensitivity of 150 times more than traditional ELISA method in the detection of interleukin-2 (IL-2). The cause for such a significant increase in sensitivity was the low sample volumes, which enabled the microfluidic device to increase the concentration and amplification of the signal from the gold particles [30].

Digital microfluidics with SPR is practical for industrial use due to its high throughput screening abilities. This combination is also efficient since there is no requirement for labelling prior to analysis. Some of the drawbacks of this integration include the potential loss of native bioactivity of immobilized molecules, non-specific adsorption and the difficulty of functionalizing metal surfaces. 

Microfluidics can also be integrated with SERS detection. This combination results in constant uniform mixing conditions; very precise and reproducible results are also obtained. The main disadvantage of this method is the difficulty in the fabrication of consistent SERS enhancer nanoparticles and the cumbersome size of Raman instruments for detection. This technique however has already been commercialized for analyte detection for homeland security in the United States and in the recognition of cancer cells [30].

## 9. Future Outlook of Biosensors

The future of biosensors looks very promising with a total yearly investment in biosensor research and development accounting for USD 300 m (€236 m) [31]. The funds spent for pathogen detection in the food industry represent $150 million per year. From a business perspective, sales of pathogen tests rose at an annual growth rate of 9.4% from $122.6 million in 2000 to $192.5 million in 2005 [32]. SPR technology has experienced extensive development as shown by the publication of 6000 biosensor research papers during the first 13 years after its introduction in the 1980s. Currently there are about 20 commercial standard SPR platforms on the market [31].

Gold nanoparticles are another innovative new approach to detecting antibiotics in food residues. Primarily, SPR signals are amplified with the use of nanoparticles and the sensor sensitivity is thereby increased [19]. Another application of gold nanoparticles involves their integration with screen-printed electrodes (SPEs). This approach offers satisfactory electrochemical properties as well as physical and chemical characteristics (extensive surface area, improved mass transport, *etc.*) due to their smaller size and versatility. This particular technique involves using Solid Phase Extraction (SPE) and integrating it with Self Assembled Monolayer (SAM) cysteine (Cys) on gold nanoparticles since Cys is very stable and densely packed. As a result, the combination of SAMs and gold electrodes increases the sensitivity and selectivity and decreases the response time [33].

The use of label-free impedimetric immunosensors is another promising alternative since the labelling process is bypassed; the method also offers excellent sensitivity and fast response. Furthermore, the sol-gel-derived silica-based material integrated with impedimetric immunosensors ensures that biorecognition elements are encapsulated at low temperatures with satisfactory mechanical and thermal stability and that they experience insignificant swelling in aqueous solution [34].

Other alternatives include the combination of immunosensor-based recognition of antibacterials with a subsequent SPR-based detection to enhance the spectrum. This technology has been applied for the immobilization of chloramphenicol in pork and of streptomycin and dihydrostreptomycin in honey, milk and meat [19]. The most recent advancement in biosensing technology is the introduction of an SPR device for portable and on-site detection of antibiotics. Immobilization techniques are based upon heptenized proteins by 1-ethyl 3-(3-dimethylaminopropyl)-carbodiimide hydrochloride/*N*-hydrosuccinimide (EDC/NHS) coupling techniques in several channels. Some of the indirect advantages of this system include the lack of a need for pre-processing of sample preparation and the good rate of repeatability with coefficient below 5% [35]. The use of portable technology is currently the most adequate approach for the detection of antibiotics in chicken muscle/blood serum due to its practicality and to the fact that its sensitivity is optimal for large-scale use in farms and slaughterhouses. However, the potential does exist to develop portable technology in order to detect a larger family of antibiotics more quickly.

## 10. Conclusions

In conclusion, antibiotic resistance is becoming recognized as a threat capable of emerging within the next 20 years. This problem is not limited to certain countries but affects the whole world with globalization acting as a catalyst. Measures have been taken by European and North American countries to set up MRL standards. However, to further reinforce the safety of meat products, there is a need to detect the excessive use of antibiotics from the source (such as farm or slaughterhouse) such that unsafe products do not reach consumers. Concerns about antibiotic resistance have piqued the interest of researchers from various disciplines. Additionally, investors have injected millions of dollars into the development of new biosensing techniques.

Presently, the most common techniques used to detect the presence of antibiotic residue are biosensor-based techniques, ELISA and liquid chromatography–mass spectrometry (LC-MS). Each of these techniques has benefits in terms of sensitivity and practicality as well as drawbacks, such as slowness and unsatisfactory cost effectiveness. This paper has provided a general overview of and comparisons between several biosensing techniques, such as electrochemical biosensors, mass-based biosensors and optical-based biosensors. There are numerous similarities and differences between biosensing mechanisms. For instance, most biosensors consist of a biorecognition element and a transducing element. However, they do not operate using the same principles or using the same recognition systems. It is to be noted that the recognition systems are equal in importance to the transducing element; both, therefore, should work in synergy for improved sensitivity. One recommended advancement would entail incorporating several biosensing techniques on one platform and using microfluidic chips to allow a merger towards the detection of multiple analytes from a single sample source. Overall, the field of biosensors consists of a broad spectrum with high potential for growth in the near future.
